# A complex intervention to improve pregnancy outcome in obese women; the UPBEAT randomised controlled trial

**DOI:** 10.1186/1471-2393-14-74

**Published:** 2014-02-18

**Authors:** Annette L Briley, Suzanne Barr, Shirlene Badger, Ruth Bell, Helen Croker, Keith M Godfrey, Bridget Holmes, Tarja I Kinnunen, Scott M Nelson, Eugene Oteng-Ntim, Nashita Patel, Stephen C Robson, Jane Sandall, Thomas Sanders, Naveed Sattar, Paul T Seed, Jane Wardle, Lucilla Poston

**Affiliations:** 1 Division of Women’s Health, Women’s Health Academic Centre, King’s College London and King’s Health Partners, 10th floor, North Wing, St.Thomas’ Hospital, London SE1 7EH, UK; 2 Division of Diabetes and Nutritional Sciences, King’s College London and King’s Health Partners, London, UK; 3 Institute of Health & Society, Newcastle University, Newcastle, UK; 4 Epidemiology and Public Health, University College London, London, UK; 5 MRC LifeCourse Epidemiology Unit, University of Southampton, Southampton, UK; 6 NIHR Biomedical Research Unit in Nutrition, Diet and Lifestyle, University of Southampton Hospital NHS Foundation Trust, Southampton, UK; 7 Centre for Population and Health Sciences, School of Medicine, University of Glasgow, Glasgow, UK; 8 Institute of Cellular Medicine, Newcastle University, Newcastle, UK

**Keywords:** Study protocol, Pregnancy, Obesity, Complex intervention, Randomised controlled trial, Glycemic index, Physical activity, Gestational diabetes, Large for gestational age

## Abstract

**Background:**

Despite the widespread recognition that obesity in pregnant women is associated with adverse outcomes for mother and child, there is no intervention proven to reduce the risk of these complications. The primary aim of this randomised controlled trial is to assess in obese pregnant women, whether a complex behavioural intervention, based on changing diet (to foods with a lower glycemic index) and physical activity, will reduce the risk of gestational diabetes (GDM) and delivery of a large for gestational age (LGA) infant. A secondary aim is to determine whether the intervention lowers the long term risk of obesity in the offspring.

**Methods/Design:**

Multicentre randomised controlled trial comparing a behavioural intervention designed to improve glycemic control with standard antenatal care in obese pregnant women.

Inclusion criteria; women with a BMI ≥30 kg/m^2^ and a singleton pregnancy between 15^+0^ weeks and 18^+6^ weeks’ gestation. Exclusion criteria; pre-defined, pre-existing diseases and multiple pregnancy. Randomisation is on-line by a computer generated programme and is minimised by BMI category, maternal age, ethnicity, parity and centre. Intervention; this is delivered by a health trainer over 8 sessions. Based on control theory, with elements of social cognitive theory, the intervention is designed to improve maternal glycemic control. Women randomised to the control arm receive standard antenatal care until delivery according to local guidelines. All women have a 75 g oral glucose tolerance test at 27^+0^- 28^+6^ weeks’ gestation.

Primary outcome; Maternal: diagnosis of GDM, according to the International Association of Diabetes in Pregnancy Study Group (IADPSG) criteria. Neonatal; infant LGA defined as >90th customised birth weight centile.

Sample size; 1546 women to provide 80% power to detect a 25% reduction in the incidence of GDM and a 30% reduction in infants large for gestational age.

**Discussion:**

All aspects of this protocol have been evaluated in a pilot randomised controlled trial, with subsequent optimisation of the intervention. The findings of this trial will inform whether lifestyle mediated improvement of glycemic control in obese pregnant women can minimise the risk of pregnancy complications.

**Trial registration:**

Current controlled trials; ISRCTN89971375.

## Background

The rise in the global incidence of obesity has reached pandemic proportions [1]. In 2008, the World Health Organisation (WHO) estimated there were 1.5 billion individuals with a body mass index (BMI) ≥ 25 kg/m^2^ including nearly 300 million obese women (BMI ≥ 30 kg/m^2^) [2]. The United Kingdom (UK) has seen a sharp increase in the proportion of obese women; as reported in a recent survey, approximately one in five women aged between 16 and 44 are obese [3]. The UK Confidential Enquiry into Maternal and Child Health identified that overweight and obesity, was either directly or indirectly the cause of over half of maternal mortality [4]. The adverse effects of obesity on reproductive health and childbearing are manifold. Obesity reduces fertility, and in pregnancy is associated with a heightened risk of gestational diabetes mellitus (GDM), hypertensive disorders of pregnancy including pre-eclampsia, and failure to progress in labour. Caesarean section rates are high, and infants of obese mothers are at greater risk of congenital malformation, large for gestational age at delivery (LGA) (>90^th^ centile), macrosomia, shoulder dystocia and stillbirth. Following delivery, obese women are more likely to suffer a postpartum haemorrhage, and have longer hospital stays than women with a normal BMI (18.5-24.9 kg/m^2^) [5,6]. The effects of obesity may extend beyond health in pregnancy; increasing evidence suggests that the children of obese women or of those whose gestational weight gain (GWG) was excessive, may be at greater risk of obesity because of antenatal exposure to adverse metabolic influences *in utero*, or in the early postnatal period [7,8].

In the UK, in contrast to the United States (US), women are no longer routinely weighed in pregnancy, except at their first antenatal appointment. The US Institute of Medicine (IOM) guidelines for weight gain during pregnancy provide recommendations for women according to their pre-pregnancy BMI, recommending that obese women should gain less weight in pregnancy (11-20 lb; 5-9 kg) than those with a lower pre-pregnancy BMI [9]. This advice is based on observational studies suggesting improved outcomes with lower weight gain. The UK National Institute for Health and Clinical Excellence (NICE) guidelines on weight management in pregnancy concluded that more evidence of improved outcomes from interventional studies is required before the US or similar guidelines for limitation of GWG are adopted [10]. Whilst review of the literature suggests that intervention studies designed to limit GWG may sometimes be effective in achieving a reduction in GWG, there is at present, no evidence for improvement of pregnancy outcome amongst obese women. However most studies, including those in overweight and obese pregnant women, have been small, not powered for clinical outcomes and have had limitations in the design [11,12].

### The role of insulin resistance in obese pregnancies

An alternative approach to restricting GWG is to focus on the adverse clinical outcomes associated with obesity, and to develop interventions which are directly associated with known underlying mechanisms. A pre-pregnancy BMI ≥30 kg/m^2^ irrespective of the amount of weight gained during pregnancy, is the most important independent determinant of the risk of caesarean section, delivery of a LGA infant and postpartum weight retention [13]. Also, the evidence linking GWG with GDM, in contrast to the strong association with pre-pregnancy BMI, is relatively weak [14]. This is, at least in part, likely to be a reflection of the strong association between maternal fat mass and insulin resistance [7]. There is a physiological increase in insulin resistance during normal pregnancy and the obese pregnant woman is at greater risk of developing GDM. Maternal hyperglycemia and, more recently, maternal hypertriglyceridemia are strongly implicated in the development of fetal macrosomia [15-18]. Using the method of continuous blood glucose monitoring, Harmon *et al.* have shown, as might be anticipated, that obese pregnant women have an exaggerated *post prandial* glucose response [17]. As the magnitude of the *post prandial* response was directly implicated in increasing fetal adiposity and birth weight through fetal hyperinsulinemia, a dietary intervention focusing on reducing *post prandial* hyperglycemia by lowering the dietary glycemic load could improve maternal glucose control, reduce the incidence of GDM and lower the incidence of LGA infants. Similarly, pre-eclampsia is associated with maternal insulin resistance, and improved glucose homeostasis might lower the risk of pre-eclampsia in obese women [19].

### Improving glycemic control in pregnancy

Specific dietary advice and increased physical activity could contribute to improved maternal glucose homeostasis [20]. In a study of 50 obese Danish women designed to limit GWG, Wolff *et al.*[21] found that an intense dietary regime (10 one-hour sessions with a dietician) focusing on healthy eating, resulted in a reduction of plasma insulin compared to women in the control arm of the study. Another study reported that a diet and exercise regime led to a reduction in GWG and a decrease in the incidence of GDM in 126 overweight and obese Australian women [22], but no difference in birth weight (3.5 kg versus 3.4 kg). In non-obese women with mild GDM, in whom improved glucose homeostasis is achieved through a strict regime of dietary intervention and insulin treatment when required, a reduction in the risk of adverse pregnancy outcome is achievable, as shown in two randomised controlled trials [23,24]. Higher levels of physical activity in normoglycemic pregnant women and those with GDM have also been shown to improve insulin sensitivity [7], but limited data of adequate power is available for the obese pregnant population. A recent meta-analysis of eight prenatal physical activity intervention studies however, showed that there was a lack of consistent evidence regarding the benefits of exercise combined or not combined with dietary advice for improving glucose tolerance in obese pregnant women, which was interpreted to reflect the limited power of current evidence and poor intervention compliance [25].

### Systematic review of the literature

Louie *et al.* conducted a systematic review of the influence of lowering dietary glycemic index (GI) in pregnancies across all BMI categories [26]. Of the eight studies included, two suggested that a low GI diet can reduce the risk of LGA infants in healthy pregnancies, but one reported an increase in small for gestational age (SGA) infants. In the three studies in which pregnancies were complicated by GDM the evidence supported the overall advantages of a low GI diet. This review recommended that until larger scale intervention trials are completed, a low GI diet should not replace the current dietary recommendations from government and health agencies, and that further research regarding the optimal time to start a low GI diet for maximum protection against adverse pregnancy outcomes is warranted.

In a systematic review of nine randomised trials including 743 overweight and obese pregnant women, Dodd *et al.* reported that there was no significant effect of interventions designed to limit GWG on weight gain or on delivery of a LGA infant [11]. In a later systematic review of thirteen randomised clinical trials of lifestyle interventions in overweight and obese pregnant women (n = 1228) we concluded that there was a modest influence on GWG (-2.21 kg; 95% confidence interval (CI) -2.86 kg to -1.59 kg), but no significant effect on any relevant clinical outcome [27]. We have also reviewed dietary and physical activity interventions in normal BMI and obese pregnant women (n = 1656 women) for the purpose of limiting GWG; in a systematic review we assessed 12 trials. Overall, diet and physical activity change was effective in reducing GWG, but there was considerable heterogeneity in outcomes [28]. The analysis highlighted differences in sample characteristics and aspects of intervention design, content, delivery and evaluation which might explain variation in effectiveness. Furthermore, failure to evaluate changes in behaviour or its psychological determinants could have obscured identification of the processes by which weight change is effective, and limited the ability to discern active intervention ingredients. We concluded that interventions should be more systematically designed and built on insights from behavioural science.

More recently, Thangaratinam *et al*., in a meta-analysis evaluated 44 clinical trials using lifestyle or dietary interventions or a combination of both during pregnancy across all BMI ranges, found a reduction in GWG (1.42 kg reduction) with any intervention in comparison to the control [12]. Physical activity alone, was associated with a reduction in birth weight (mean difference -60 g, 95% CI -120 g to -10 g). Interventions based on diet were the most effective, being associated with reductions in maternal GWG (3.84 kg, 95% CI 2.45 to 5.22 kg) and a modest improvement of obstetric outcomes. However, the combination of intervention methods did not result in a reduction in the incidence of LGA between the groups (RR 0.85, 95% CI 0.66 to 1.09). Amongst obese women, there was no evidence for an improvement of any clinical outcome. In an editorial, to this review, we highlighted that there remains a paucity of information regarding intensity, duration and compliance of the interventions, all of which could account for the lack of efficacy, as well minimal evidence for any effect of the intervention on the targeted behaviours. If the intervention does not achieve a change of behaviour in the anticipated direction, it follows that there will be no influence on clinical outcomes [29].

The protocol presented here describes a complex behavioural intervention comprising dietary and physical activity changes which we have developed with the aim of improving glycemic control in obese pregnant women. The intervention is based on established control theory with elements of social cognitive theory [30,31]. The primary hypothesis being tested is that an antenatal intervention package of low glycemic dietary advice combined with advice on increased physical activity will reduce the incidence of maternal GDM and LGA infants. A secondary hypothesis is that the intervention will reduce the risk of obesity in the child. Prior to undertaking a trial adequately powered to investigate clinical outcomes, we completed a pilot study (n = 183 women) to determine whether the intervention changed dietary and physical behaviours as anticipated [32]. This pilot study showed that diet but not physical activity (as objectively measured) changed with the intervention and that all aspects of the protocol were feasible. A process evaluation led to optimisation of intervention delivery. The trial steering committee recommended continuation with recruitment for the randomised controlled trial (RCT), and it was decided that the physical activity aspect of the intervention should remain, as this follows standard guidelines for pregnant women [33].

## Methods/Design

### Study design

Multicentre RCT. For participating centres see the UPBEAT trial website: http://www.medscinet.net/upbeat/.

### Ethical approval

NHS Research Ethics Committee approval was obtained in all centres (UK IRAS integrated research application system; reference 09/H0802/5). *Inclusion Criteria:* Women with a singleton pregnancy, 15^+0^- 18^+6^ weeks’ gestation and body mass index ≥30 kg/m^2^ at first antenatal appointment.

#### Exclusion criteria

Women unable or unwilling to give informed consent; <15^+0^ weeks or >18^+6^ weeks’ gestation; essential hypertension requiring treatment either pre-pregnancy or in index pregnancy; pre-existing renal disease; systemic lupus erythematosus; antiphospholipid syndrome; sickle cell disease; thalassemia; coeliac disease; thyroid disease; current psychosis; multiple pregnancy; currently prescribed metformin.

**Figure 1:**
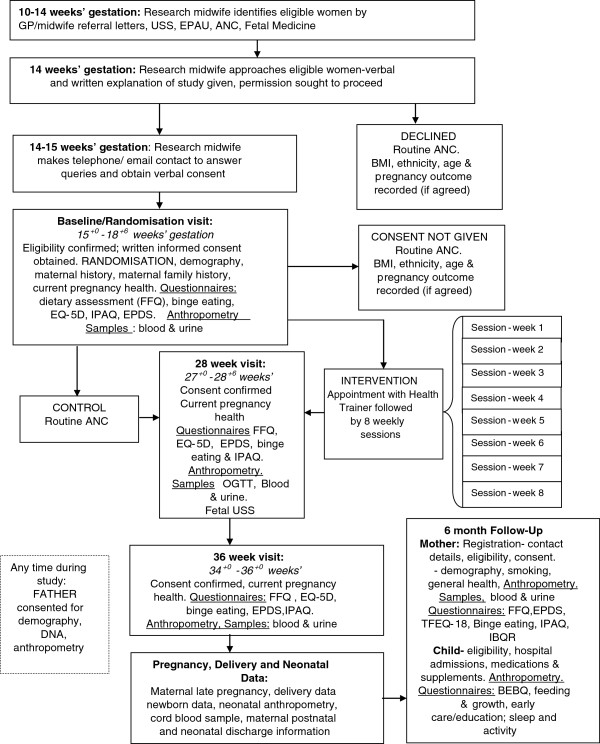
**UPBEAT protocol summary.** Abbreviations: ANC; Antenatal clinic, BEBQ; baby eating behaviour questionnaire, BMI; Body mass index, DNA; Deoxyribonucleic acid, EPAU; Early pregnancy assessment unit, EPDS; Edinburgh postnatal depression score questionnaire. EQ-5D; EuroQuol 5 dimension quality of life questionnaire FFQ; Food frequency questionnaire, GP; General Practitioner, IBQ-R: Infant behaviour questionnaire revised; IPAQ; International physical activity questionnaire; LGA; Large for gestational age OGTT; Oral glucose tolerance test, SGA; Small for gestational age, TFEQ-18; Three-factor eating questionnaire-R18, USS; Ultrasound scanning.

The protocol for the study is shown in Figure 1.

### Trial entry

Eligible women are identified in antenatal clinics and from general practitioner and midwives referral letters. Verbal and written information is given. Research midwives contact potential recruits, obtain verbal consent and arrange the first appointment. For those who decline to participate, permission is sought to collect minimal pregnancy outcome data.

### 15^+0^-18^+6^ weeks’ appointment: baseline and randomisation

At the first appointment, written informed consent is obtained. Baseline demography, medical and family history and current pregnancy information is collected. A short validated food frequency questionnaire (FFQ) [34] is completed to evaluate dietary glycemic load, dietary glycemic index, saturated fat and total sugar intake and other dietary variables. Women are weighed, pulse and blood pressure are checked, anthropometric measurements obtained and blood and urine samples taken. Behavioural and psychological measures include; the EuroQuol Quality of life (EQ-5D) [35], the Edinburgh Postnatal Depression Scale (EPDS) [36], the International Physical Activity Questionnaire (IPAQ) [37], and a ‘binge eating’ screening questionnaire [38]. Randomisation occurs at this visit via a secure internet based data management system (MedSciNet™), which is the repository for all trial data. The randomisation schedule is minimised according to ethnicity, parity (0 *versus* ≥ 1), age, BMI (BMI 30-34.9 kg/m^2^*versus* 35-39.9 kg/m^2^ and >40 kg/m^2^) and centre. Randomised women are allocated sequential study numbers, regardless of allocation to the intervention or standard care group.

### Intervention

Women randomised to the intervention group attend a one-to-one interview with the health trainer, which includes discussion of the potential benefits of attending the weekly sessions. In the UK, health trainers help people to change their behaviour to achieve personal choices and goals, and generally do not have pre-specified health professional qualifications, but relevant experience. All health trainers in this trial receive study-specific training in all aspects of the intervention and ongoing support throughout the trial. Women in the intervention group receive a participant handbook, a DVD of an exercise regime safe for pregnancy, a pedometer and a logbook for recording weekly SMART (Specific, Measurable, Achievable, Relevant and Time specific) goals, and steps as assessed by pedometer. They are invited to attend 8 sessions with the health trainer on a weekly basis, each lasting 1 to 1.5 hours. Women are encouraged to attend all sessions, but are strongly recommended to attend a minimum of five. For the sessions not attended in person, the health trainer covers the session material by phone or email. Attendance and coverage of session material are documented in the study database. Following a review of the dietary and physical intervention, each session is designed to focus on different approaches in achieving the goals set. These include SMART goals, self monitoring, and provision of feedback regarding goal attainment, identification and problem solving of barriers, enlisting social support and providing opportunities for social comparison. At each session, review of the previous weeks’ goals is undertaken.

The dietary intervention aims to promote a healthier pattern of eating, similar to that used in diabetes prevention studies, but does not aim to restrict energy intake. In order to decrease the glycemic load, dietary advice includes exchanging starchy foods with a medium/high GI for those with a lower dietary GI, and restricting the consumption of sugar-sweetened beverages (including fruit juice) but not fruit. Participants are also given dietary advice to reduce saturated fatty acid intake.

Advice regarding physical activity focuses on increasing the daily step count incrementally, and being more active in daily life. Pedometers are used for monitoring and motivation. The emphasis is on walking at a moderate intensity with additional options included, especially for those who are already engaging in some physical activity. This degree of activity accords with that recommended by the UK Royal College of Obstetricians and Gynaecologists [33].

### Standard care

Women randomised to the standard care group attend routine antenatal care according to local health care provision. UK recommendations state that women with a BMI ≥ 30 kg/m^2^ should be advised by a health professional at the earliest opportunity of the risks of obesity in pregnancy and be given advice about a healthy diet and safe levels of physical activity. Recommendations for referral to a registered dietician are infrequently implemented. Women are only weighed at their first antenatal visit [10].

### 27^+0^- 28^+6^ weeks’ appointment

All women in both groups attend for an oral glucose tolerance test (OGTT) at 27^+0^- 28^+6^ weeks’ gestation (fasting for a minimum of 10 hours, 75 g glucose load). At this visit weight and anthropometric measurements are taken, health in current pregnancy noted, additional blood and urine samples taken, dietary FFQ, EQ-5D, EPDS, IPAQ and ‘binge eating’ questionnaires completed. Early pregnancy data including blood pressure, blood chemistry and anomaly scan reports are entered from routine clinical records.

### 34^+0^- 36^+0^ weeks’ appointment

Women in both arms of the study attend the research appointment at 34^+0^- 36^+0^ weeks’ gestation. Current health in pregnancy is recorded, weight and anthropometric measurements taken, blood and urine samples collected and dietary FFQ, EQ-5D, EPDS, IPAQ and ‘binge eating’ questionnaires completed.

Unexpected adverse events are reported in accordance with good clinical practice guidance.

### Pregnancy outcome data

Following delivery, information is collected from maternal medical records regarding health in late pregnancy, labour onset, mode of delivery, blood loss, antenatal and postnatal inpatient nights. Where possible a cord blood sample is taken.

Neonatal and postnatal outcome data includes Apgar scores, admission to special care baby unit and inpatient nights. To address the influence of the intervention on fetal growth and adiposity, neonatal anthropometry and length measurements are undertaken within 72 hours of birth.

### Six months postpartum

To determine whether the intervention has led to sustained change in maternal dietary and physical activity behaviours, diet is assessed by FFQ and physical activity by IPAQ. Maternal demographic data, health since pregnancy and smoking history is obtained. Maternal anthropometric measures are taken. EPDS, Three Factor Eating Questionnaire R18 (TEFQ-18) [39] and ‘binge eating’ questionnaires are completed. To address safety and the influence of the intervention on the long term health of the child, details regarding the child’s health from birth are obtained. If cord blood was not taken, and if the parents provide consent, a buccal cell sample is taken from the child’s mouth for DNA extraction (Oragene™). To address the potential influence of the intervention on infant adiposity at 6 months and obtain information on known determinants of childhood obesity, infant length and other anthropometric measures are taken. The mother provides information for an infant feeding and growth questionnaire [40] and a validated questionnaire addressing appetite (Baby Eating and Behaviour Questionnaire (BEBQ) [41]. Information on activity using questions from the Infant Behaviour Questionnaire -Revised (IBQ-R) [42] and sleep patterns are obtained [43] and information on childcare (kindergarten, other carers) collected.

### Paternal data

At any point during the pregnancy or at the 6 month postnatal appointment the father of the baby is asked to consent to taking part in the study to provide information which may influence the health of the child. A brief medical history, blood pressure and pulse are checked, anthropometric measurements taken and a blood samples collected for the provision for DNA. In the absence of direct paternal measurement, women are asked to recall their partner’s height and weight and brief medical and smoking history.

### Study endpoints

#### Primary maternal outcome

GDM diagnosed by OGTT at 27^+0^ – 28^+6^ weeks’ gestation according to the criteria recommended by IADPSG. Diagnosis of GDM; fasting capillary glucose ≥ 5.1 mmol/L and/or 1 hour glucose ≥10 mmol/L and/or 2 hour glucose ≥8.5 mmol/L [44].

#### Primary neonatal outcome

LGA delivery defined as adjusted birth weight >90^th^ centile [45] for gestational age adjusting for maternal height, corrected maternal weight, ethnicity, parity, and sex of baby.

#### Secondary outcomes

*Maternal* - Pre-eclampsia, severe pre-eclampsia; mode of delivery: caesarean section (elective, emergency, pre-labour, in labour), vaginal delivery, operative vaginal delivery; induction of labour; blood loss at delivery (>1000 ml; >2000 ml); inpatient nights (antenatal, postnatal); GWG, trimester specific GWG; fasting plasma glucose, fasting plasma insulin, insulin resistance calculated by homeostatic model assessment 2 (HOMA2-IR) [46] at 28 weeks’ gestation; diagnosis of GDM by local criteria; referral to GDM antenatal service following OGTT; requirement for insulin or metformin during pregnancy; fetal growth at 28 weeks. Health related quality of life as assessed by EQ-5D. At 27^+0^- 28^+6^ and 34^+0^- 36^+0^ weeks’ gestation and 6 months postpartum; mid-arm, neck, hip, thigh and wrist circumference and skinfold thickness (subscapular, triceps, biceps, supra-iliac); plasma fructosamine, triglycerides, LDL, VLDL and HDL cholesterol, plasma insulin, C-reactive protein, other relevant epigenetic and metabolomic biomarkers, and urinary biomarkers; dietary measures including glycemic load, saturated fat and total sugar intake; dietary feeding patterns; physical activity scores; measures of depression; maternal smoking. At 6 months postpartum, postnatal weight retention, existing maternal morbidity (diabetic status, hypertension, thromboembolism, low mood [47]).

*Neonatal;* Gestational age at delivery, delivery at <37 weeks’, delivery at <34 weeks’; birth weight, birth weight >4,000 g, <2,500 g; birth weight >95^th^ <10th and <5^th^ customised birth weight centile, distribution of birth weight, neonatal death, days in special care baby unit, total inpatient days, need for mechanical ventilation and duration, discharge home on O_2_, suspected and confirmed infection, evidence of intraventricular haemorrhage and other complications, (pulmonary haemorrhage, necrotizing enterocolitis), retinopathy of prematurity, hypoglycaemia. Occipitofrontal head circumference, abdominal circumference, mid-arm circumference, chest circumference, crown-rump length and crown-heel length (neonatometer), triceps and subscapular skin fold thicknesses and estimated fat mass.

Key epigenetic and metabolomic biomarkers will be investigated using cord blood or whole blood (maternal and fetal) and their relation to specific outcomes.

*Infant at 6 months*; duration of breast feeding, choice of formula milk, weaning history (introduction of foods and frequency/timing of foods), a general measure of appetite, and four specific scales: enjoyment of food, food responsiveness, slowness in eating, and satiety responsiveness, anthropometric measurements (occipitofrontal circumference, abdominal circumference, mid-arm circumference, chest circumference, crown-rump length and crown-heel length by infantometer, subscapular and triceps skin fold thicknesses and estimated fat mass; activity (total number of 14 standard milestones reached) and sleeping patterns (time spent sleeping; morning, afternoon and night; health care resource use (hospital admissions and medications); frequency of use of kindergarten/mother’s help.

### Sub-group analysis

Women who are treated for GDM; differences in diagnostic thresholds between centres will be accommodated by minimisation by centre. Other subgroups likely to be of interest include demographic and socio-economic status (assessed by Index Multiple Deprivation), ethnic groups, BMI categories, groups of different parity and smokers.

Interaction tests will be used to determine whether treatment is particularly effective in individual subgroups. Performance of subgroup analysis will be dependent on sufficient data. Because of the well known risk of false positives, both main effects and interaction tests will be performed before considering results for subgroups.

#### Sample size

In the pilot RCT [32], 30% of women in the standard care arm developed GDM according to the IADPSG criteria [39]. 1546 women (including allowance for 20% drop out) (773/arm) will be recruited to provide 80% power to detect a 25% reduction in the incidence of GDM. Considering LGA deliveries, for a 30% relative risk reduction (RRR) from an estimated 17.2% of LGA to 12.0% in the intervention arm; 1546 women would give 80% power [48,49].

#### Analysis

To determine whether the trial participants are representative of the general population, relevant parameters available from electronic summary patient records will be compared between eligible women agreeing and declining to take part. Analyses will follow the intention-to-treat principle.

Following CONSORT guidelines, risk ratios and risk differences will be estimated by binary regression for Yes/No outcomes. Where measurements are repeated over time, results [mean (SD) or n (%)] will be presented separately at each time point. Randomised comparisons with 95% confidence intervals will be made using linear regression with robust standard errors, adjusting for the baseline value where appropriate.

Multiple regression models will be used to address the influence of maternal exposures on neonatal and infant (6 months) body composition and the role of paternal factors.

## Discussion

This RCT will determine whether a complex intervention addressing diet and physical activity will reduce the incidence of GDM and LGA infants in a population of obese pregnant women receiving antenatal care in the UK. The study will inform guidelines on the management of obesity in pregnancy, and if successful, is designed to be rapidly transferrable to clinical practice. Determination of infant anthropometry at 6 months of age will assess whether the intervention in pregnancy can influence body composition of the infant. Further studies on childhood body composition at 3 years of age will also be undertaken.

## Abbreviations

BMI: Body mass index; FFQ: Food frequency questionnaire; EPDS: Edinburgh Postnatal Depression Scale; EQ-5D: EuroQuol-5D; GDM: Gestational diabetes mellitus; GI: Glycemic index; GL: Glycemic load; GWG: Gestational weight gain; HOMA2-IR: Homeostatic Model Assessment-Insulin resistance; IADPSG: International Association of Diabetes Pregnancy Study Group; IPAQ: International Physical Activity Questionnaire; LGA: large for gestational age; O2: Oxygen; OGTT: Oral glucose tolerance test; PE: Pre-eclampsia; RCT: Randomised controlled trial; RRR: Relative risk reduction; SGA: Small for gestational age; SMART (goals): Specific, Measurable, Achievable, Relevant and Time-Specific.

## Competing interests

None of the authors disclosed any financial or non-financial competing interests.

## Authors’ contributions

LP, ALB and KMG contributed to all aspects design of the study and LP and ALB had overall responsibility for the study. LP, ALB, NP drafting of the manuscript; BH, TS and SB(Barr) contributed to development of the dietary elements of the intervention; RB, TIK and SCR contributed to the development of the physical activity elements of the intervention; HC and JW contributed to development of the behavioural elements of intervention, SMN and NS contributed to assessment of maternal glucose homeostasis and biochemical analyses; EO-N contributed to the development of the final protocol; JS and SB(Badger) through process evaluation of the pilot trial contributed to development of the final protocol; PTS developed the data analysis protocol. All authors were involved in drafting of the manuscript and gave approval for the final version of the manuscript.

## Authors’ information

LP is Professor of Maternal and Fetal Health, TS is Professor of Nutritional Science, SB(Badger) a Research Associate and SB(Barr) a Research Dietician at King’s College London; RB is a Senior Lecturer in Public Health Medicine, and SCR Professor of Fetal Medicine at Newcastle University; KMG is Professor of Epidemiology & Human Development and Director Centre for the Developmental Origins of Health and Disease at Southampton University; SMN is Professor of Reproductive and Maternal Medicine, and NS Professor of Metabolic Medicine at Glasgow University; EO-N is a Consultant Obstetrician and ALB a Consultant Midwife at Guy’s and St. Thomas’ NHS Foundation Trust, London; PTS is a Senior Lecturer in Medical Statistics at King’s College London and JW is Professor in Clinical Psychology and Director of the Health Behaviour Unit at University College London. TIK, HC and NP are research fellows.

## Pre-publication history

The pre-publication history for this paper can be accessed here:

http://www.biomedcentral.com/1471-2393/14/74/prepub
