# Erratum: A complex intervention to improve pregnancy outcome in obese women; the UPBEAT randomised controlled trial

**DOI:** 10.1186/s12884-015-0540-1

**Published:** 2015-05-08

**Authors:** Annette L Briley, Suzanne Barr, Shirlene Badger, Ruth Bell, Helen Croker, Keith M Godfrey, Bridget Holmes, Tarja I Kinnunen, Scott M Nelson, Eugene Oteng-Ntim, Nashita Patel, Stephen C Robson, Jane Sandall, Thomas Sanders, Naveed Sattar, Paul T Seed, Jane Wardle, Lucilla Poston

**Affiliations:** Division of Women’s Health, Women’s Health Academic Centre, King’s College London and King’s Health Partners, 10th floor, North Wing, St.Thomas’ Hospital, London, SE1 7EH UK; Division of Diabetes and Nutritional Sciences, King’s College London and King’s Health Partners, London, UK; Institute of Health & Society, Newcastle University, Newcastle, UK; Epidemiology and Public Health, University College London, London, UK; MRC LifeCourse Epidemiology Unit, University of Southampton, Southampton, UK; NIHR Biomedical Research Unit in Nutrition, Diet and Lifestyle, University of Southampton Hospital NHS Foundation Trust, Southampton, UK; Centre for Population and Health Sciences, School of Medicine, University of Glasgow, Glasgow, UK; Institute of Cellular Medicine, Newcastle University, Newcastle, UK

## Erratum

After publication of the original article [1] it came to the publishers attention that pre-existing diabetes had been omitted from the exclusion criteria in the manuscript. The paragraph on page 4 should read:

*Exclusion criteria*

Women unable or unwilling to give informed consent; <15 + 0 weeks or >18 + 6 weeks’ gestation; essential hypertension requiring treatment either pre-pregnancy or in index pregnancy; pre-existing diabetes (Type 1 or Type 2); pre-existing renal disease; systemic lupus erythematosus; antiphospholipid syndrome; sickle cell disease; thalassemia; coeliac disease; thyroid disease; current psychosis; multiple pregnancy; currently prescribed metformin.

We apologize for any inconvenience this has caused.
